# Supplementary material for: Ultrasound-promoted synthesis of 2-organoselanyl-naphthalenes using Oxone® in aqueous medium as an oxidizing agent
Source: PeerJ. 2018 May 7;6:e4706. doi: 10.7717/peerj.4706 (PMC5944430; doi:10.7717/peerj.4706)
Supplement: Supplemental Information 1 [file peerj-06-4706-s001.docx]

**Ultrasound-promoted synthesis of 2-organoselanyl-naphthalenes using Oxone^®^ in aqueous medium as oxidizing agent**

Gelson Perin*^1^, Daniela Rodrigues Araujo^1^, Patrick Carvalho Nobre^1^, Eder João Lenardão*^1^, Raquel Guimarães Jacob^1^, Márcio Santos Silva^2^ and Juliano Alex Roehrs^3^

^1^ Laboratório de Síntese Orgânica Limpa – LASOL, Universidade Federal de Pelotas – UFPel, Pelotas, RS, Brazil.

^2^ Centro de Ciências Naturais e Humanas – CCNH, Universidade Federal do ABC – UFABC. Santo André, SP, Brazil.

^3^ Instituto Federal de Educação Ciência e Tecnologia Sul-rio-grandense – IFSul, Pelotas, RS, Brazil.

*Corresponding authors: lenardao@ufpel.edu.br (E. J. Lenardão) and

gelson_perin@ufpel.edu.br (G. Perin)

**Selected Spectra:**

**Selected Spectra**

**Figure 1S**. ^1^H NMR (400 MHz, CDCl_3_) of the product **3a**.

**Figure 2S**. ^13^C NMR (100 MHz, CDCl_3_) of the product **3a**.

**Figure 3S**. ^1^H NMR (400 MHz, CDCl_3_) of the product **3b**.

**Figure 4S**. ^13^C NMR (100 MHz, CDCl_3_) of the product **3b**.

**Figure 5S**. ^1^H NMR (400 MHz, CDCl_3_) of the product **3c**.

**Figure 6S**. ^13^C NMR (100 MHz, CDCl_3_) of the product **3c**.

**Figure 7S**. ^1^H NMR (400 MHz, CDCl_3_) of the product **3d**.

**Figure 8S**. ^13^C NMR (100 MHz, CDCl_3_) of the product **3d**.

** Figure 9S**. ^1^H NMR (400 MHz, CDCl_3_) of the product **3e**.

**Figure 10S**. ^13^C NMR (100 MHz, CDCl_3_) of the product **3e**.

**Figure 11S**. ^1^H NMR (400 MHz, CDCl_3_) of the product **3f**.

 ****

**Figure 12S**. ^13^C NMR (100 MHz, CDCl_3_) of the product **3f**.


**Figure 13S**. ^1^H NMR (400 MHz, CDCl_3_) of the product **3g**.

 ****

**Figure 14S**. ^13^C NMR (100 MHz, CDCl_3_) of the product **3g**.

**Figure 15S**. ^13^C NMR (400 MHz, CDCl_3_) of the product **3h**.

**Figure 16S**. ^13^C NMR (100 MHz, CDCl_3_) of the product **3h**.

**Figure 17S**. ^1^H NMR (400 MHz, CDCl_3_) of the product **3i**.

**Figure 18S**. ^13^C NMR (100 MHz, CDCl_3_) of the product **3i**.
